# Supporting breast cancer screening decisions for caregivers of older women with dementia: study protocol for a randomized controlled trial

**DOI:** 10.1186/s13063-018-3039-z

**Published:** 2018-12-12

**Authors:** Nicole R. Fowler, Mara A. Schonberg, Greg A. Sachs, Peter H. Schwartz, Sujuan Gao, Kathleen A. Lane, Lev Inger, Alexia M. Torke

**Affiliations:** 1 Indiana University School of Medicine, 1101 West 10th Street, Indianapolis, IN 46202 USA; 2 Division of General Internal Medicine and Geriatrics, 1101 West 10th Street, Indianapolis, IN 46202 USA; 3 Regenstrief Institute, Indiana University Center for Aging Research, 1101 West 10th Street, Indianapolis, IN 46202 USA; 4 Sandra Eskenazi Center for Brain Care Innovation, 1101 West 10th Street, Indianapolis, IN 46202 USA; 5 Division of General Medicine and Primary Care, Beth Israel Deaconess Medical Center, 330 Brookline Avenue, Boston, MA 02215 USA; 6 Center for Bioethics, 1101 West 10th Street, Indianapolis, IN 46202 USA; 7 Department of Biostatistics, 410 W. 10th Street, Suite 3000, Indianapolis, IN 46202 USA

**Keywords:** Alzheimer’s disease, breast cancer, dementia, mammography, screening

## Abstract

**Background:**

Alzheimer’s disease and related dementias (ADRD) impact a woman’s life expectancy and her ability to participate in medical decision-making about breast cancer screening, necessitating the involvement of family caregivers. Making decisions about mammography screening for women with ADRD is stressful. There are no data that suggest that breast cancer screening helps women with ADRD live longer or better. Decision aids may improve the quality of decision-making about mammography for ADRD patients and may inform family caregivers about the risks, benefits, and need for decision-making around mammography screening.

**Methods/design:**

The Decisions about Cancer Screening in Alzheimer’s Disease (DECAD) trial, a randomized controlled clinical trial, will enroll 426 dyads of older women with ADRD (≥75 years) and a family caregiver from clinics and primary-care practices in Indiana to test a novel, evidence-based decision aid. This decision aid includes information about the impact of ADRD on life expectancy, the benefit of mammograms, and the impact on the quality of life for older women with ADRD. Dyads will be randomized to receive the decision aid or active control information about home safety. This trial will examine the effect on the caregiver’s decisional conflict (primary outcome) and the caregiver’s decision-making self-efficacy (secondary outcome). A second follow-up at 15 months will include a brief, semi-structured interview with the caregiver regarding the patient’s experience with mammograms and decision-making about mammograms. At the same time, a review of the patient’s electronic medical record (EMR) will look at discussions about mammography with their primary-care physician and mammogram orders, receipt, results, and burden (e.g., additional diagnostic procedures due to false-positive results, identification of an abnormality on the screening exam but further work-up declined, and identification of a clinically unimportant cancer). A third follow-up at 24 months will extract EMR data on mammogram orders, occurrences, results, and the burden of mammograms.

**Discussion:**

We hypothesize that caregivers who receive the decision aid will have lower levels of decisional conflict and higher levels of decision-making self-efficacy compared to the control group. We also hypothesize that the DECAD decision aid will reduce mammography use among older women with ADRD.

**Trial registration:**

Clinical Trials Register, NCT03282097. Registered on 13 September 2017.

**Electronic supplementary material:**

The online version of this article (10.1186/s13063-018-3039-z) contains supplementary material, which is available to authorized users.

## Background

The incidence of both Alzheimer’s disease and related dementias (ADRD) and breast cancer increases with age [1, 2]; thus, many older women with ADRD are faced with questions about breast cancer screening [3, 4]. Some current recommendations for breast cancer screening use life expectancy (e.g., age, functional status, and co-morbidity status) to form their guidelines on the clinical appropriateness of mammography [5–12] (http://www.choosingwisely.org/clinician-lists/amda-cancer-screenings-if-life-expectancy-less-than-10-years/). The data suggest that women need a life expectancy of 10 years to have a mortality benefit from mammography screening. Some guidelines specifically recommend not screening women with a life expectancy <10 years [5, 6, 8, 9, 11–15] (http://www.choosingwisely.org/clinician-lists/society-general-internal-medicine-cancer-screening-in-adults-with-life-expectancy-less-than-10-years/) and note that screening beyond 75 years of age should be approached with caution due to the increased burden and risk of overdiagnosis and overtreatment [7, 8, 11, 16]. The average life expectancy of older women with ADRD is <10 years [16–21], and approximately 26% of women with ADRD (over 800,000 in the U.S. population) receive screening mammograms annually at an average annual cost of $32 million [2, 22] (http://www.choosingwisely.org/clinician-lists/society-general-internal-medicine-cancer-screening-in-adults-with-life-expectancy-less-than-10-years/). While some women with ADRD discontinue mammograms as they age or become more impaired, many continue, even following a serious health event, such as a myocardial infarction or stroke, or well beyond the time that it is clinically beneficial [3, 4, 15, 19, 22–25] (http://www.choosingwisely.org/clinician-lists/amda-cancer-screenings-if-life-expectancy-less-than-10-years/).

The risks, burdens, and harms of mammography screening for older women with ADRD are both physical and psychological and encompass medical interventions often referred to as the treatment cascade [26, 27]. Screening women with ADRD can potentially put them at risk of physical and psychological harm as a result of overdiagnosis, overtreatment, additional tests due to false positives, and the identification of a clinically unimportant cancer [4, 25, 28, 29]. Women with ADRD may also experience additional burdens because of their cognitive impairment. For example, these women may not understand the purpose of the test or become confused or agitated when waiting or having the test conducted [22, 25, 30, 31]. The test itself has been reported by caregivers as more burdensome for older women with ADRD [4, 32]. Conversely, while mammography screening may not help older women with ADRD live longer, it may help find breast cancers earlier when they are easier to treat [33]. Therefore, it is important that women with ADRD and their caregivers have all the pertinent information needed to make an informed decision about screening.

Because ADRD can impact a woman’s ability to participate in her medical decision-making about breast cancer screening [31, 34, 35], family and non-paid friend caregivers (hereafter referred to as caregivers) are frequently involved in making decisions about mammography [3, 4, 32, 36]. Caregivers report stress, decisional conflict, and a lack of knowledge about and confidence in making mammography decisions for their relative with ADRD [3, 4, 28, 37], which can increase their burden [35, 36, 38]. Additionally, they cite concerns that screening tests may continue due to scheduled reminders or habit, even when the test becomes more burdensome and less beneficial to the patient [4, 28]. Informational tools, such as decision aids, that describe the proximate risks and distant benefits [39] of mammography based on an individual’s life expectancy, comorbidity status, and personal preference may reduce caregiver decisional conflict and decision-making self-efficacy, and facilitate screening discussions between ADRD caregivers and primary-care physicians (PCPs) [40–46]. For example, Hanson et al. [43] created and tested a decision aid for surrogates of nursing home residents with ADRD to support caregiver decisions about feeding options. They found that a decision aid decreased caregivers’ decisional conflict, improved their knowledge scores, and enhanced discussions with providers about feeding options. These studies support the potential impact of decision aids on improved ADRD patient and caregiver outcomes.

To fill the gap in providing ADRD patients and their caregivers with decision support about mammography screening, we created a new decision aid that is written for ADRD caregivers and is specific for older women with ADRD. The design of this decision aid was informed by a decision aid on mammography screening for older women without dementia developed by Dr. Mara Schonberg (co-investigator) [47, 48], our pilot work [28, 32], and critera established for the development and testing of evidence-based decision aids [49]. Our research team is now conducting a randomized controlled trial, Decisions about Cancer Screening in Alzheimer’s Disease (DECAD), to test if this new evidence-based decision aid can improve the quality of decision-making about mammography in older women with ADRD. The primary outcome is a caregiver-assessed measure of decisional conflict about breast cancer screening. Secondary outcomes include other measures of decision quality and utilization of mammograms. We hypothesize that caregivers of patients who use the decision aid will have lower levels of decisional conflict as measured by the decisional conflict scale (DCS) and higher levels of decision-making self-efficacy as measured by the decision self-efficacy scale (DSE) compared to the control group. We also hypothesize that the DECAD decision aid will reduce mammography use among older women with ADRD within the follow-up period of 24 months.

## Methods/design

### DECAD study design

The DECAD study is a multi-center randomized controlled trial of an evidence-based mammography screening decision aid for older women with ADRD and their caregivers. It will enroll 426 ADRD patient/caregiver dyads; 213 dyads will be randomized to receive the decision aid and 213 dyads will receive an active control in the form of a pamphlet on home safety. This study protocol follows the Standard Protocol Items: Recommendations for Interventional Trials (SPIRIT) Guidelines (Additional file 1). The trial will be conducted and reported according to the reporting of pragmatic trials: an extension of the Consolidated Standards of Reporting Trials (CONSORT) Statement. The study has been approved by the institutional review board of Indiana University (1501278953A022). The DECAD trial is registered with clinicaltrials.gov (NCT03282097).

### Setting and study population

Patient/caregiver dyads for DECAD will be recruited from the following sites, which serve diverse populations of patients throughout Indiana, USA: the Indiana University Alzheimer Disease Center; the Aging Brain Care Program at Eskenazi Health (EH); primary-care sites at EH; the Departments of Medicine, Neurology, and Psychiatry at Indiana University Health (IUH); primary-care sites affiliated with IUH; support groups for ADRD caregivers hosted by the Alzheimer’s Association-Greater Indiana Chapter; and other organizations that support persons with ADRD. Recruitment in the clinical sites will occur via the Indiana University Practice-Based Research Network (IU-PBRN), which covers research recruitment in all primary-care practices affiliated with EH and IUH [50]. All primary-care practices affiliated with EH are federally qualified health centers. The Aging Brain Care Program combines a specialty clinic and home-based dementia collaborative care program serving patients with ADRD and other cognitive disorders. In the state of Indiana, IUH has more than 200 primary-care providers and 150,000 patients, and it receives 330,000 annual primary-care visits. The Indiana University Alzheimer Disease Center is one of 32 centers funded by the National Institute on Aging that support ADRD research in the country. Recruitment via the Alzheimer’s Association support groups and other community organizations will be facilitated through the Indiana University Center for Aging Research Community Engagement Initiative [51].

### Eligibility

The target population is dyads of caregivers of women aged 75 years or older with a diagnosis of ADRD and the woman with ADRD. The eligibility of patients will be established through screening the Indiana Network for Patient Care (INPC) database or the local practice medical record. For caregivers, assessments will be conducted by research assistants face-to-face or via the telephone.

#### Inclusion criteria

The following inclusion criteria apply to the patients: female, ≥75 years, at least one mammogram in the past 5 years, a primary-care visit scheduled in the next 12 months, diagnosis of ADRD as determined by the ICD-10 code in their ambulatory electronic health record, ability to provide informed consent or assent, and ability to communicate in and read English. Determination of capacity for the patient to consent is assessed prior to obtaining consent by trained study personnel using the teach-back method with questions specific to the risks and benefits of participating in the study [52]. The caregiver inclusion criteria are as follows: 18 years or older; primary family, friend, or non-paid caregiver of the patient identified by the patient, self-identified, or listed as the primary caregiver in the electronic medical record (EMR) or registry; ability to provide informed consent; and ability to communicate in and read English.

#### Exclusion criteria

The following exclusion criteria apply to the patients: permanent resident of a nursing facility; had a mammogram in the past 9 months; primary-care visit scheduled is the first visit with the PCP; has already decided to stop getting mammograms; history of atypical ductal hyperplasia, lobular carcinoma in situ, ductal carcinoma in situ, or other invasive breast cancer in the past five years; diagnosis of mild cognitive impairment or serious mental illness, such as bipolar disorder or schizophrenia, as determined by the ICD-10 code. The caregiver exclusion criteria are as follows: less than a 7th grade education; has already decided that the patient will stop getting mammograms; and diagnosis of ADRD or a serious mental illness, such as bipolar disorder or schizophrenia, as determined by the ICD-10 code.

### Recruitment

Dyads will be recruited throughout the state of Indiana using the IU-PBRN, the local EMR systems, research registries at Indiana University, and advertising. Data generated by the IU-PBRN will be collected and stored in the INPC, which is the central Indiana Regional Health Information Exchange [53]. Given that not all patients will have a caregiver living with them during the study and that caregiving arrangements will vary, we will identify the person who is the primary caregiver or informant and ensure that they attend the patient’s next primary-care visit. Rolling enrollment will take place over 36 months with a planned average monthly enrollment of 11 or 12 dyads.

Data managers of the INPC and local health-care systems will identify eligible patients based on the inclusion criteria (i.e., any woman ≥75 years of age with a diagnosis of ADRD who has received a mammogram in the past 5 years, who does not have a 5-year history of breast cancer, and for whom it is not recorded in her EMR that she has decided to stop mammography). The details of eligible patients recruited from a clinical site will be provided to the patient’s PCP. Following approval by the PCPs, study personnel will approach eligible dyad participants via the telephone to confirm interest and eligibility and to obtain informed consent. Those being recruited from research registries will be contacted directly and without prior approval from their PCP since they have already provided consent to be contacted for research. We will contact the primary caregiver for each eligible woman. Caregivers will be eligible if they are the only or are one of the primary caregivers for the patient and intend to accompany the patient to the next clinic visit.

### Randomization

The unit of randomization will be the patient/caregiver dyad. Dyads will be randomly assigned in a 1:1 ratio to one of two groups (mammogram decision aid or pamphlet on home safety) and stratified by recruitment site to control for institutional effects and the styles of different PCPs.

Study statisticians will use a computer-generated randomization scheme to assign dyads, rather than providers or clinics, to the intervention or control group to minimize the effects of unmeasured case mix differences and clinic-level clustering. Based on descriptive data from the recruitment sites, the large number of individual recruitment sites, and the total number of PCPs, the risk of spillover from having participating dyads interact or PCPs treat patients who have caregivers in both the intervention and control group is likely to be small. Additionally, we found from our pilot work that intervention materials are unlikely to be shared with others, which leaves the control dyads unexposed [28].

### Description of the intervention

Based on a mammography screening decision aid designed by Dr. Schonberg [47, 48], the investigative team designed a decision aid that is specific for older women with ADRD and their caregivers [28, 32]. The wording, context, and content of the decision aid reflect the literature on the risks and benefits of mammography screening for older women with ADRD [24, 45, 49, 54]. Specifically, the decision aid is written at a 6th grade reading level and includes text and visual information on: (1) breast cancer risk factors for women >75 years, (2) health and life expectancy with ADRD, (3) likely outcomes if screened and not screened with mammography, (4) competing mortality risks, (5) breast cancer treatments, (6) an acknowledgment of the emotions of caregivers who participate in medical decision-making for their loved one with ADRD, and (7) tips on how to talk with the patient’s PCP about mammograms.

### Description of the control

To reduce response bias and to compensate for the time and attention required by the intervention group to read the decision aid, caregivers in the control arm will be given a two-page pamphlet on home safety for older adults developed by the Foundation for Health in Aging of the American Geriatrics Society. The pamphlet contains tips about important actions older adults can take to safeguard themselves from falls, poisoning, bathroom hazards, abuse, fire, and other home-related hazards [55].

We do not plan any intervention for the PCPs because we do not want to change their usual behavior. However, if PCPs request a copy of the educational materials then we will email them a copy of the home safety pamphlet following the post PCP assessment.

### Delivery of intervention and assessments

**Fig. 1:**
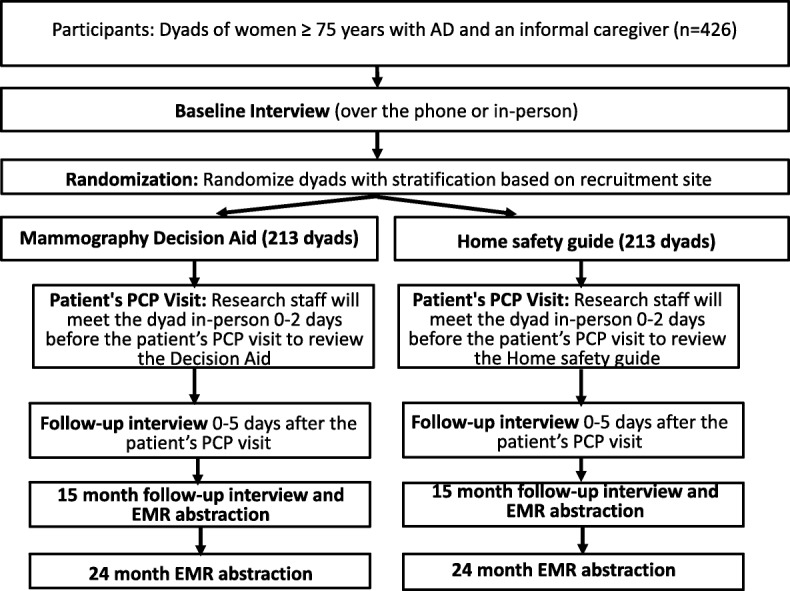
Flowchart of study participation and intervention

Both members of the dyad will receive the decision aid or pamphlet on home safety by mail following enrollment, baseline data collection, and randomization (Fig. 1). A letter accompanying the decision aid or pamphlet on home safety requests that the caregiver review the decision aid or pamphlet on home safety on their own and with the patient before the patient’s next PCP visit. A trained research assistant will then meet with the dyad together and in-person 0–2 days prior to the index PCP visit. At that encounter, the research assistant will ensure that the caregiver has reviewed all pages of the decision aid or pamphlet on home safety and, if needed, supply a second copy if either member of the dyad did not bring it with them. The research assistant will not discuss the content of the decision aid or pamphlet on home safety with the caregiver; they will simply ensure that they have read it in its entirety before the patient’s PCP visit. If the caregiver or patient has any questions about the content of the decision aid or pamphlet on home safety, the research assistant will encourage them to ask the patient’s PCP or to discuss the materials with the PCP. If the index PCP visit is canceled, the dyad will remain enrolled and will be followed until the visit is rescheduled. If the visit is never rescheduled during the study window, the dyad will remain in the study in their assigned group for an intention-to-treat analysis.

The follow-up with the dyad after the PCP visit (conducted in phone or in-person by a research assistant) will ideally be scheduled immediately after the PCP visit, but no later than 5 days after the PCP visit to measure post-visit outcomes. Caregiver assessments at this time include decisional conflict, decision-making self-efficacy, their role in decision-making, their intentions for whether the patient will be screened, their knowledge of mammography screening in older women with ADRD, and what they discussed about mammography with the PCP at the index visit (continue, discontinue, or no decision). Patient assessments at the post-visit follow-up include their role in decision-making and their intentions for whether they will be screened.

A second follow-up at 15 months will be conducted and will include a brief semi-structured interview with a trained research assistant. At this time, patient data will be extracted from their EMR on: what they discussed about mammography with the PCP at any visit since the index visit (continue, discontinue, or no decision), orders for mammograms, receipt of a mammogram, data on the mammogram results if received, and data on the burden of mammograms (e.g., additional diagnostic procedures due to false-positive results, identification of an abnormality on the screening exam but further work-up declined, identification of a clinically unimportant cancer, and documentation of depressive symptoms, anxiety, or pain related to the screening experience). The semi-structured interviews will assess the caregiver’s perceptions about the patient’s experience with mammograms, if received, and about any decisions that they and the patient have made about mammograms.

The third and final 24-month follow-up of EMR data will be conducted to measure mammogram orders, receipt, results, and burden.

### Primary and secondary outcome measures

All outcomes and measures for the DECAD trial are summarized in Table 1. The primary outcome will be assessed using the DCS to determine the impact of the decision aid on caregivers’ decisional conflict about whether their relative with ADRD should receive a mammography. The DCS includes 16 questions regarding a medical decision that they have made or that they are about to make [56]. It is a validated and widely accepted measure of decision quality that has been used in previous studies of decision aids intended for ADRD caregivers [57, 58]. On average, the DCS takes 5–7 min to complete. Test-retest correlations and Cronbach alpha coefficients exceed 0.78, and tests of predictive validity found that for every one unit increase in the DCS, people were 59 times more likely to change their mind, 23 times more likely to delay their decision, and 5 times more likely to express regret about a decision [56, 59–62].

**Table 1:** Outcomes and measures

Outcomes	Outcome measures	Description	Scoring	When	Source
Primary outcome	Decisional conflict	16-items on a 1–5-point Likert scale. Measures uncertainty around a decision, whether they feels informed, clear about their personal values, and supported in their decision-making [55]	Scores range 0–100; lower scores indicate less conflict	Baseline, follow-up	Caregiver reported
Secondary outcomes	Decision-making self-efficacy	11 items on a 5-point Likert scale. Measures of self-confidence or belief in their ability to make decisions [62]	Scores range 0–100; higher scores indicate more self-efficacy	Baseline, follow-up	Caregiver reported
	Intention to be screened	Three items. Two items assess propensity to get person with ADRD screened. One item asks how many more mammograms they think the patient will get	Yes vs. those who are unsure or plan not to be screened	Baseline, follow-up	Caregiver reported; patient reported
	Receipt of screening	Discussion with caregiver	Yes vs. no	15-month follow-up	Caregiver reported
		Review primary-care notes, radiology reports, and documentation on screening and preventive care; caregiver report.	Yes vs. no	15- and 24-month follow-ups	Patient EMR
	Knowledge	16-items (6 multiple choice and 10 true/false) [48]	Sum of correct answers	Baseline, follow-up	Caregiver reported
	Burden of screening on patient	Review patient’s EMR for additional diagnostic procedures due to false-positive results, identification of an abnormality on screening exam but further work-up declined, identification of a clinically unimportant cancer; documentation of depressive symptoms, anxiety, or pain related to the screening experience	Yes or no	15- and 24-month follow-ups	Patient EMR
	Burden of screening on caregiver	Measure perceptions about the burden of the mammography for the patient; semi-structured questions about patient’s mammogram experience and perceived burden of screening	Descriptive	15-month follow-up	Caregiver reported; patient reported
	Role in decision-making	Assesses preferences for and involvement in making decisions on their own or sharing responsibility with their family or doctor [71]	Active vs. passive/shared with doctor (since aim of decision aids is to help dyads be more active in decision-making)	Follow-up	Caregiver reported; patient reported
	Acceptability of the materials	Assess caregivers’ and patients’ perceptions about the length, clarity, and helpfulness of the decision aid and their willingness to recommend it. The number of times they reviewed it, how many pages they read, how long it took them to read it, how they would prefer to receive it if not part of a study	Descriptive	Follow-up	Caregiver reported; patient reported

Secondary outcome measures include decision-making self-efficacy, receipt of a mammogram, caregiver knowledge about mammograms for older women with ADRD, and the patient’s and caregiver’s role in the mammography decision. To measure decision-making self-efficacy, we will use the DSE, a validated 11-item instrument that measures how confident the respondent is in their ability to make an informed medical decision [63]. We will use the DSE to measure caregiver decision-making self-efficacy for mammography decisions for their relative with ADRD. To measure the change in self-efficacy over time in both groups, we will measure it at two time points [63]. In previous studies, the psychometric properties of the DSE had an alpha coefficient of 0.92 [64].

Other measures include the intention to be screened as well as the burden of screening on the patient and caregiver. Intentions to be screened will be examined using one item on a 5-point scale and another categorical item that asks how many more mammograms the patient and caregiver think that the patient will get in her lifetime. We will categorize scores as 1–2 (no), 3 (unsure), or 4–5 (yes) [48, 65]. Knowledge about mammograms for older women with ADRD will be measured with a 16-item measure mapped directly from the information presented in the decision aid [48]. We also will collect data on the role of the patient and caregiver in the decision about mammography, if a decision occurred, and assess the extent to which each member of the dyad participated in the review of the decision aid through the semi-structured interviews. This will allow us to describe differences in the roles of the patient and caregiver in decision-making among different dyads.

Perceptions of the burden of screening on the caregiver will be determined using structured questions about the mammogram experience. Additionally, the burden of screening on the patient will be evaluated by reviewing the patient’s EMR for additional diagnostic procedures due to false-positive results; identification of an abnormality on the screening exam but further work-up declined; identification of a clinically unimportant cancer; and documentation of depressive symptoms, anxiety, or pain related to the screening experience.

### Data monitoring

The data safety monitoring plan for this trial will be monitored by the principal investigator and a four-member data safety and monitoring board. The board’s charter contains a detailed list of the board’s responsibilities. The board will act in an advisory capacity to the institutional review board and a National Institute on Aging program official and it will monitor participant safety and evaluate the progress of the study. It will review the procedures for maintaining the confidentiality of the data and review the quality of data collection, management, and analyses.

Potential adverse events, such as new breast cancer diagnoses, complications of receiving a mammogram, complications from surgical or medical treatment following an abnormal mammogram, or distress related to receiving a mammogram, will be monitored continually by the DECAD research manager and discussed weekly by the research team. All adverse events and unanticipated problems will be reported to the study’s principal investigator within 24 h. If unanticipated serious adverse events occur (i.e., those not of a type listed in the data and safety monitoring plan) that are related to the intervention, they will be reported within 48 h of the study team’s learning of them.

### Data collection

Data for the DECAD trial will be collected at baseline, at the follow-up after the index PCP visit, and at 15 and 24 months following the intervention. Following the confirmation of eligibility and after providing informed consent, the caregivers in both arms will complete the baseline assessment, which includes the DCS and DSE, intention to allow screening, severity of cognitive impairment using the dementia severity rating scale [66], knowledge [48], health literacy based on the short test of functional health literacy in adults (S-TOFHLA) [67], and numeracy based on the subjective numeracy scale [68]. Patient baseline assessments include intention to undergo screening, and their attitudes, norms, and experiences with mammograms. Research staff who administer the follow-up assessments will be blinded to dyad intervention status and they will be trained to read all questionnaires verbatim and not to add commentary.

In addition to the primary and secondary measures, we will collect social and demographic data on all patients and caregivers, including age, sex, and race. We also will collect the relationship of the caregiver to the patient, frequency and type of contact with the patient, geographic distance from the patient, education level, annual income, self-reported health status, severity of cognitive impairment with the dementia severity rating scale [66], the degree of functional impairment, and how bothersome or upsetting that impairment is to the caregiver using the caregiver assessment of function and upset tool [69]. Additional descriptive data regarding the patient will be obtained from recruitment site databases and the INPC including, but not limited to, date of ADRD diagnosis and co-morbidities.

Data will be collected face-to-face at the clinic, in the caregivers’ or patients’ home, or via telephone. Data will also be obtained from the EMR. All survey data will be entered into a database using Research Electronic Data Capture (REDCap), a secure web application available through our Clinical and Translational Science Institute [13].

### Timeline

**Fig. 2:**
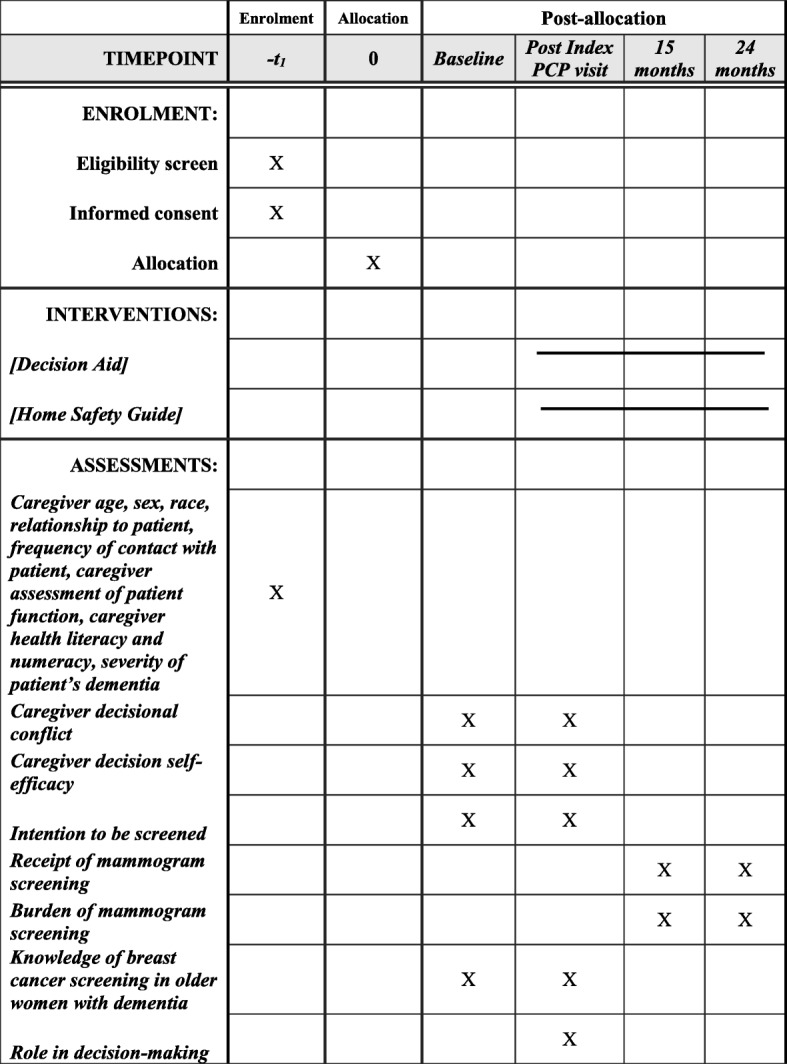
Schedule of enrollment, interventions, and assessments

The recruitment of patients started on 1 December 2017 and is expected to be finalized by December 2020. All data from all follow-ups is expected to be collected by 2022. The data analysis, writing of scientific manuscripts, and submissions to peer-reviewed scientific journals will be carried out during 2021, 2022, and 2023. Figure 2 is the schedule of enrollment, interventions, and assessments.

### Analysis plan

#### Study arm comparison

We will examine univariate distributions of continuous variables to detect any potential violations of the assumptions in our planned parametric method of analysis. We will transform variables as needed to ensure normal distribution assumptions are met. We will use nonparametric methods if the transformations are inadequate. Th demographic characteristics of the patients and caregivers will be compared to evaluate if the two groups are effectively balanced. We will use chi-squared tests or Fisher’s exact tests to compare the frequencies of categorical variables. Analysis of variance (ANOVA), or its nonparametric alternative, the Wilcoxon rank sum test, will be used to compare the distribution of continuous variables between the groups.

Analysis of covariance (ANCOVA) models will be used to compare mean DCS scores between the intervention and control groups. For each caregiver, the difference in DCS scores from baseline to follow-up will be used as the dependent variable in ANCOVA with randomization group assignment as the independent variable and other potential baseline covariates that are found to be significantly different between the two groups in the univariate comparisons. ANCOVA models will also be used to compare changes in decision-making self-efficacy and intention to screen at follow-up between the intervention and control groups after adjusting for site and other potential baseline covariates found to be significantly different between the two groups in the univariate comparisons.

A binary indicator for receipt of mammograms within 24 months from the intervention date will be tested in a logistic regression model. The group (decision aid or control) and other potential baseline covariates found to be different in the univariate comparisons will be used as the independent variable.

Subgroup analyses will be used for both the primary and secondary outcomes, examining any potential moderating effect of patients’ and caregivers’ characteristics on decision aid impact. Likelihood ratio tests in mixed effect models will be used to determine whether there are significant clustering effects due to physicians. Mixed effects adjustments for physician effects will be used in the presence of significant clustering effects. The following patient variables will be included in subgroup analyses: age, level of cognitive impairment, level of functional impairment, number of co-morbidities, and their role in mammography decisions. The following caregiver variables will be included in subgroup analyses: relationship to the patient, level of education, level of bother or upset by patient functional impairments, and perceived burden of mammography for the patient and for them at baseline. We will include each of these variables in the ANCOVA models for the primary outcome (caregiver decisional confect) and in the logistic models for the secondary outcomes (caregiver decision-making self-efficacy, intention to screen, and patient receipt of mammogram), and test for interactions between these variables and the intervention group adjusting for recruitment sites and other potential baseline covariates. Significant interaction between a patient/caregiver characteristic variable and group would indicate different intervention effects in the dyad subgroups defined by the variable. We will use SAS 9.4 for all analyses (SAS Institute, Carey, NC).

An analysis of the semi-structured qualitative data from the 15-month caregiver assessments will begin after each assessment is completed to refine the data collection process and to identify and pursue emergent themes for subsequent caregiver interviews. Interviews will be conducted by telephone, unless the caregiver requests an in-person interview, and will be audio-recorded and transcribed verbatim. Two independent coders will code each set of responses and a three-step coding process derived from the sociological tradition of grounded theory will be used to analyze the recorded interviews [70]. Data from the qualitative interviews will enhance the findings from the primary and secondary outcomes of the trial by adding nuanced information from the caregiver about how they used the decision aid, details of any discussions or decisions about mammograms, their experience of the patient receiving a mammogram, and their perception of the patient’s experience in arranging to have a mammogram.

#### Justification of sample size

The sample size for the proposed trial was calculated using our pilot data, which showed effect sizes of 0.28 on DCS score and 0.34 for self-efficacy scores. To detect an effect size of 0.28 or greater on DCS scores between the two groups, we will need to have 202 patient/caregiver dyads per group to complete both baseline and follow-up with 80.2% power using a two-sample *t-*test at 0.05 level. Allowing for 5% missing data at the follow-up, we will need to enroll 213 patient/caregiver dyads per group (total enrollment of 426). With our planned sample size, we will have 92.6% power to detect an effect size of 0.34 or greater on changes in self-efficacy scores between the two groups.

Using EMR data from the recruitment sites in this trial, we identified 9588 women who are ≥75 years and who had had a mammogram in the last 5 years, resulting in an estimated mammogram screening rate of 27.5% in our patient population. In detecting a change in the uptake of mammograms, 213 patient/caregiver dyads per group will have 80% power to detect a 42% reduction in the screening rate (27.5% in the control group and 15.9% in the decision aid group) using Fisher’s exact test at α = 0.05. For the exploratory aim, assuming equal sample sizes in the two subgroups and screening rates in the control group stay at 27%, we will have 80% power to detect a screening rate of 11.5% (a reduction of 58%) in one subgroup and a screening rate of 22% (reduction of 20%) in the other subgroup using Fisher’s exact test at 0.05 significance level.

## Discussion

The benefits and harms of mammograms for older women are not known, as no randomized controlled trial has included women with ADRD who are ≥75 years [23, 37, 71]. In part because of this lacuna, guidelines on mammography screening in older women vary [5–12] (http://www.choosingwisely.org/clinician-lists/amda-cancer-screenings-if-life-expectancy-less-than-10-years/). The DECAD study is the only randomized controlled trial of a decision aid to support ADRD caregivers in deciding on cancer screening, which is a common decision in primary care. If the DECAD trial is successful, the results may also inform the development of other decision aids for ADRD caregivers in primary-care settings, addressing other cancer screening tests, such as a colonoscopy, or other treatments of questionable value for patients with ADRD.

The DECAD study is methodologically innovative in that it will test a new paradigm for shared decision-making that involves the patient/caregiver dyad. Specifically, the proposed work will extend the shared decision-making of the caregiver to a dynamic model that is responsive to the communication and decision-making needs of the surrogates of patients with ADRD. As part of the DECAD study, we will measure both the caregivers’ role and the patients’ role in decision-making about mammograms and the burden of mammograms from their perspective. Also, we will test the effect of ADRD severity on the use of the decision aid. Results from DECAD will be extended to help us learn how to present the significant risks of medical interventions, in general, to both ADRD patients and caregivers, which is essential for providing high-quality cost-effective care.

The DECAD trial has some limitations. The intervention is intended to promote and enhance shared decision-making between caregivers of women with ADRD and the women’s PCP, but the study does not interact directly with PCPs. We considered an alternative design where both the caregiver and the PCP receive the decision aid but decided to measure the decision aid’s effectiveness on the patient/caregiver dyad since the decision aid was designed for caregivers. If we detect a significant clustering effect due to physician, we will analyze the data using mixed effects models to adjust for physician clustering. This will lead to a lower power than what we presented in our power estimates. Additionally, regarding possible contamination, there may be situations where some of the content in our decision aid is made available to dyads in the control group through interactions with physicians or through other channels. However, our decision aid presents specific and focused data about mammograms in the context of older women with ADRD, and our trial is designed to detect differences in outcomes between the two randomized groups. In situations with moderate contamination rates, it has been shown that individualized randomization can be more efficient in terms of sample size compared to cluster randomization [72].

To minimize any impact, we will stratify randomization by site. Lastly, given the nature of the intervention, blinding of the dyad is not possible. However, research assistants who collect post-PCP outcome assessments are blinded to each dyad’s allocation and are trained to administer the assessments the same to all dyads and without any indication of randomization group.

In conclusion, DECAD is an evaluation of a decision aid intervention for ADRD caregivers with high potential to be implemented into primary care. Given the inherent complexities of caring for persons with ADRD in primary care and the frequency of caregiver participation in medical decisions, we anticipate that the use of decision aids will facilitate quality decisions regarding mammography screening. The decrease in the use of cancer screening tests in some patients with ADRD will reduce the burden on both the patient and caregiver and change the practice and behavior with respect to cancer screening. The results from this study will inform other interventions that support ADRD caregivers’ decision-making around other types of medical care and treatment and that are tailored to match the ADRD patient’s life expectancy and goals of care.

### Trial status

This protocol was registered in ClinicalTrial.gov under identifier NCT03282097 on 13 September 2017 and the last update was made on 21 November 2017. The current protocol is version 2, dated 24 May 2018. Recruitment started on 1 December 2017 and is expected to be complete by December 2022. This trial is currently in the patient selection and intervention stages. To date, 100 patient/caregiver dyads have been enrolled, none of whom have completed the trial.

## Additional file

Additional file 1: SPIRIT 2013 Checklist: Recommended items to address in a clinical trial protocol and related documents. (DOC 119 kb)

## Abbreviations

ADRD: Alzheimer’s disease and related dementia
ANCOVA: Analysis of covariance
ANOVA: Analysis of variance
CONSORT: Consolidated Standards of Reporting Trials
DCS: Decisional conflict scale
DECAD: Decisions about Cancer Screening in Alzheimer’s Disease
DSE: Decision self-efficacy scale
EH: Eskenazi Health
EMR: Electronic medical record
INPC: Indiana Network for Patient Care
IUH: Indiana University Health
IU-PBRN: Indiana University Practice-based Research Network
PCP: Primary-care physician
SPIRIT: Standard Protocol Items: Recommendations for Interventional Trials
